# Surgical treatment of primary cardiac tumors in children: Experience of a single institute

**DOI:** 10.3892/ol.2015.3529

**Published:** 2015-07-23

**Authors:** CHENGMING FAN, ZIBO GAO, NI YIN, JINFU YANG

**Affiliations:** Department of Cardiothoracic Surgery, The Second Xiangya Hospital, Central South University, Changsha, Hunan 410011, P.R. China

**Keywords:** cardiac tumor, primary, surgery

## Abstract

In order to review the surgical experience of a single institute with regard to the treatment of primary cardiac tumors, data was collected on patients with a histopathological diagnosis of a primary cardiac tumor (with the exception of myxoma) in a retrospective analysis of those treated between 2004 and 2013. In total, 11 patients were identified, with a mean age at diagnosis of 23 months and a mean weight of 13 kg. The most frequent cause of referral was a cardiac murmur and the most frequent cardiac tumor was rhabdomyoma (5 cases), followed by fibroma (3 cases), angiofibroma (2 cases) and fibrosarcoma (1 case). Surgical removal of the tumor was performed in all patients due to the respective clinical symptoms. A subtotal resection was performed in a single patient (with angiofibroma invading the aortic root, superior vena cava and sinus node) due to financial constraints. This patient succumbed to tumor invasion 2 years later. Another of the patients (pericardial fibrosarcoma) succumbed 1 year after the total resection, as they were unable to undergo a repeat surgery for the relapsed tumor due to financial inadequacy. The remaining 9 patients have survived in good condition during the 1–6 year follow-up. Surgery is the preferred treatment for patients with symptomatic primary cardiac tumors, and has good early- and long-term outcomes. However, due to the current health care system inadequacies in China, certain parents cannot afford the medical expenses, thus, more comprehensive social security and medical insurance may require consideration.

## Introduction

Primary cardiac tumors, which can occur at any age, are extremely rare in children, with an incidence varying between 0.0017 and 0.28%, with >90% of these cases being benign (1). Primary cardiac tumors in children may arise in any region of the myocardium, endocardium or pericardium (1). The most common cardiac tumor is rhabdomyoma (67%), usually associated with tuberous sclerosis, followed by fibroma (17%) and fibroelastoma (8%). Clinical presentation is dependent on the age of the patient, as well as the size and location of the cardiac tumor (2). Children with cardiac tumors can be asymptomatic or may present with symptoms such as, murmur, arrhythmia, heart failure, or sudden death (2–4). Biopsy is the diagnostic gold standard (1). Treatment modalities include conservative management, surgical resection and transplantation, and treatment choice varies according to the diagnosis and the patient's symptoms (2). Surgery is the main approach for patients with symptomatic primary cardiac tumors (3,4). Due to the rarity of theprimary cardiac tumors, literature regarding the management and outcome remains limited (5). However, low coverage is a major problem with the current social security and medical insurance in China. In the present study, a retrospective analysis was performed on the medical records of patients with a diagnosis of primary cardiac tumor (with the exception of myxoma) who were treated between 2004 and 2013. A total of 11 patients were identified. This study describes the experience of a single institute with regard to these cases of primary cardiac tumors in infants and children.

## Patients and methods

### 

#### Clinical data

**Figure 1.**
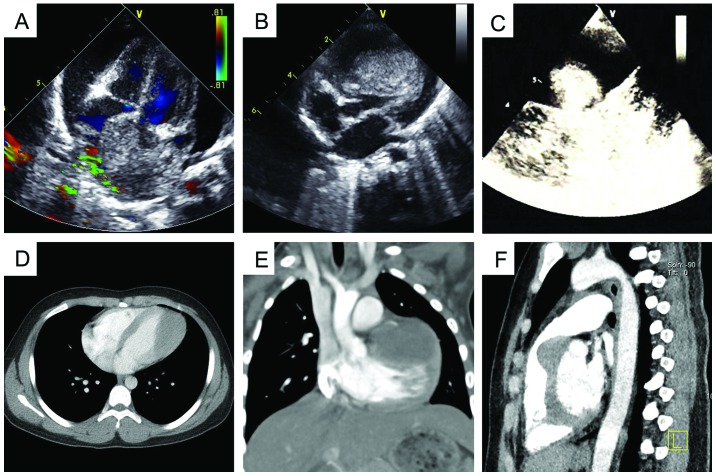
Echocardiography and computed tomography angiography prior to surgery. (A) Tumor in the atrial septum with moderate pericardial effusion. (B) Right ventricular tumor with outflow tract obstruction. (C) Intrapericardial mass. (D) Left ventricle tumor. (E) Left ventricle tumor with left coronary artery involvement. (F) Right ventricular tumor.

Between May 2004 and March 2013, 11 children (9 males and 2 females) diagnosed with primary cardiac tumors underwent a tumor resection at the Second Xiangya Hospital of Central South University (Changsha, Hunan, China). Patient age ranged between 13 days and 4 years (mean, 23 months), with a mean weight of 13±5.7 kg. The most common clinical symptom was a cardiac murmur, followed by intolerance to feeding, palpitations and dyspnea. Chest X-ray showed mild to moderate cardiac enlargement (10 cases) and pulmonary oligemia (4 cases). Echocardiography revealed masses inside two cardiac chambers (Fig. 1A), a mass inside the right ventricle (Fig. 1B) and an intrapericardial mass (Fig. 1C). Examination by cardiac computed tomography also revealed cardiac masses (Fig. 1D-F). Written informed consent was obtained from the patients' families prior to publication of the data.

#### Surgical procedure

All surgeries in this study were performed via a median sternotomy. Patients with intrapericardial masses underwent excision off-pump, while the remaining patients were placed on cardiopulmonary bypass (CPB). In 1 patient, a tumor was found involving the aortic root, superior vena cava (SVC) and sinus node after opening the pericardium, thus, a total tumor resection with aortic root replacement, SVC patch repair and implantation of a permanent cardiac pacemaker was indicated. However, following communication with the patient's parents, the surgery was refused and a partial tumor resection was requested due to insufficient financial support. Thus, a partial resection and pathological biopsy were performed. All other patients underwent a total tumor resection successfully. The associated cardiac deformities were also corrected at the same time. A patient with a ventricular septal defect (VSD), in whom the diagnosis of a primary cardiac tumor was missed pre-operatively, was treated, and a mass was found behind the left ventricular free wall during surgery. VSD patch repair and a total resection of the tumor were subsequently performed.

## Results

**Figure 2.**
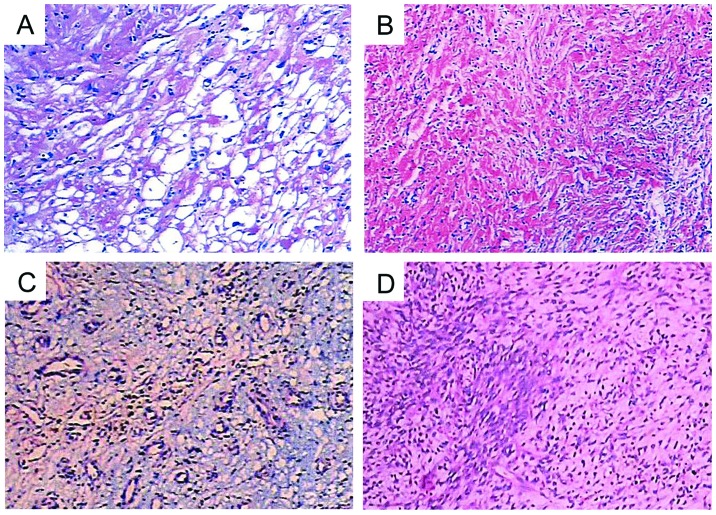
Post-operative histological examination showing (A) rhabdomyoma, (B) fibroma, (C) angiofibroma and (D) fibrosarcoma. Hematoxylin and eosin staining. Magnification, ×100.

Rhabdomyoma (Fig. 2A), fibroma (Fig. 2B), angiofibroma (Fig. 2C) and fibrosarcoma (Fig. 2D) were histologically diagnosed in 5, 3, 2 and 1 patient, respectively. The tumor involving the aortic root, SVC and sinus node was found to be an infiltrating angiofibroma. The patients were discharged on post-operative days 7–10 without any complications, including pericardial effusion, arrhythmia and pulmonary embolism. Echocardiography at 1 week post-surgery showed normal blood flow velocities in the atrioventricular and aortic valve orifices. At the 6-month follow-up, the patient who underwent a partial resection of a diffuse infiltrating angiofibroma was diagnosed with a residual tumor, 32 mm in diameter, which infiltrated the aortic root and SVC upon three-dimensional echocardiography. Further surgery was refused and the patient succumbed to the disease 2 years after the initial surgery. For the patient in whom cardiac tamponade was initially identified, a pericardial fibrosarcoma was diagnosed following a total resection performed as an emergency procedure. Echocardiography shown tumor recurrence 1 year after the resection. However, the patient's custodian refused a repeat surgical procedure and the patient succumbed 1 year after the initial surgery. The remaining 9 patients who underwent complete tumor resections are alive and well at 2–9 years of follow-up, and show no signs of tumor relapse.

## Discussion

Primary cardiac tumors may be malignant or benign, and may arise in any region of the myocardium, endocardium or pericardium (1). The clinical symptoms are based on the tumor location, number, size and shape, as well as the nature of the tumor. The tumors vary from being completely asymptomatic to presenting with arrhythmia, tumor embolism, severe angina or sudden mortality (2–4). Timely surgical removal of the tumor is necessary when the patient shows associated symptoms. Myxoma, as with thrombosis, was excluded from the present study owing to its higher incidence and its relatively fixed surgical options.

Surgery is recommended for patients with symptomatic primary cardiac tumors. Thus, once diagnosed, a total tumor resection should be performed to avoid the increasing surgical risk with tumor progression. Once the tumor has progressed to a non-resectable life-threatening stage, one remaining treatment option is a cardiac transplant (6). Rhabdomyoma is the most common primary cardiac tumor in children. Rhabdomyomas tend to regress spontaneously, and close observation must be ensured in patients without symptoms (7). Surgery is required in cases with severe symptoms, such as obstruction in the ventricular output tracts (3). Cardiac fibromas are associated with high mortality rates, and spontaneous regression rarely occurs. Surgical resection is normally recommended in infants due to the high risk of lethal arrhythmias, right ventricular outflow tract obstruction or heart failure (4). Angiofibroma tends to involve the cardiac valves, and these tumors should be removed in a timely manner (8). A previous study with one of the largest patient groups studied by autopsy recorded an incidence rate of 0.0022% for primary pericardial tumors (9). Primary pericardial fibrosarcoma in children is extremely rare, and surgery is required when the diagnosis has been confirmed.

Primary cardiac tumors, with the exception of certain regions of pericardial tumors, should be removed with the affected patients on CPB. One surgical principle is that the preservation of remaining heart function should be the first priority, with total resection as the second. If a resection of the tumor is not achievable in severely symptomatic patients, such as those with a large tumors affecting the left side of the heart or in the case of a malignant tumor, cardiac transplantation may be considered. To date, there have been no evidence-based studies on whether post-operative radiotherapy and/or chemotherapy is necessary for the treatment of primary pericardial sarcomas (10).

In the present study, all patients underwent surgical removal of the tumor. Two patients succumbed during the follow-up period. Not everyone in the Chinese population is covered by health insurance schemes, particularly with regard to medical insurance for serious illnesses, and with the tense doctor-patient relationship in China, this results in surgeons having to undertake more secure and economical procedures, rather than the most optimal treatment. Healthcare must be pushed step by step, and it is also important to improve the morale and working environment of health professionals (11). The main goal should be for every single Chinese patient to obtain the most optimal treatment in the near future.

In conclusion, individualized treatment should be introduced for children with primary cardiac tumors. Surgery is required in symptomatic cases, and has good early and long-term outcomes. For those Chinese parents who would prefer to procreate again rather than pay for the treatment of such affected patients, more education and comprehensive Social Security and Medicare may require consideration.
